# Diagnostic Tools and Biomarkers for Severe Drug Eruptions

**DOI:** 10.3390/ijms22147527

**Published:** 2021-07-14

**Authors:** Manabu Yoshioka, Yu Sawada, Motonobu Nakamura

**Affiliations:** Department of Dermatology, University of Occupational and Environmental Health, Kitakyushu 807-8555, Japan; manabu-yoshioka@med.uoeh-u.ac.jp (M.Y.); motonaka@med.uoeh-u.ac.jp (M.N.)

**Keywords:** drug eruption, Stevens-Johnson syndrome, toxic epidermal necrolysis, biomarker, diagnosis

## Abstract

In accordance with the development of human technology, various medications have been speedily developed in the current decade. While they have beneficial impact on various diseases, these medications accidentally cause adverse reactions, especially drug eruption. This delayed hypersensitivity reaction in the skin sometimes causes a life-threatening adverse reaction, namely Stevens-Johnson syndrome and toxic epidermal necrolysis. Therefore, how to identify these clinical courses in early time points is a critical issue. To improve this problem, various biomarkers have been found for these severe cutaneous adverse reactions through recent research. Granulysin, Fas ligands, perforin, and granzyme B are recognized as useful biomarkers to evaluate the early onset of Stevens-Johnson syndrome and toxic epidermal necrolysis, and other biomarkers, such as miRNAs, high mobility group box 1 protein (HMGB1), and S100A2, which are also helpful to identify the severe cutaneous adverse reactions. Because these tools have been currently well developed, updates of the knowledge in this field are necessary for clinicians. In this review, we focused on the detailed biomarkers and diagnostic tools for drug eruption and we also discussed the actual usefulness of these biomarkers in the clinical aspects based on the pathogenesis of drug eruption.

## 1. Introduction

The current advancement of various technologies gives us many beneficial advantages for living in this world, as an abundance of chemicals, molecules, and medications have been developed [1,2]. These materials can accidentally influence the human body, creating disadvantages for human health, with the representative adverse reaction being allergies against these materials, sometimes causing a critical life-threatening reaction in the human body [3,4,5]. Immunity in peripheral organs regulates host defense function against external environmental stimuli. Skin is a representative peripheral lymphoid organ, which is located on the most outside in the human body and is well exposed to external stimuli and antigens [6,7,8]. Some harmful external antigens provoke cutaneous immune response mediated by Type IV delayed hypersensitivity reaction [9]. This is one of the major pathogenesis of drug eruption, which sometimes develops into severe cutaneous drug adverse reactions, such as Stevens-Johnson syndrome (SJS) and toxic epidermal necrolysis (TEN), which increases the risk of medication-induced-death [10]. In the current treatment, there is a limited number of impact treatments against these severe cutaneous adverse reactions. Therefore, it is important for clinicians to identify the detection at an early stage in severe cutaneous drug adverse events and the useful biomarkers for severe cutaneous adverse events [11]. It is also important to run a pharmacovigilance program in order to detect new associations among drugs and new cutaneous reactions early on [12]. This review focused on the biomarker and diagnostic tools for the detection of severe cutaneous adverse events and other minority types of drug eruption by searching keywords, “drug eruption”, “biomarker”, and “symptom” using Pubmed. These findings will be helpful for the further direction of these fields in the future.

## 2. Pathogenesis of Drug Eruption

**Figure 1:**
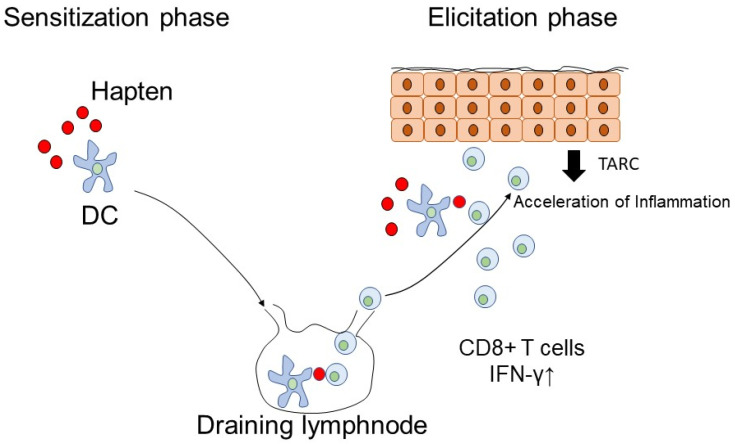
The mechanism of delayed hypersensitivity reaction in the skin. In the mechanisms of delayed hypersensitivity reaction, there are two phases, namely, the sensitization phase and the elicitation phase. In the sensitization phase, after external antigen exposure, antigen presentation cells pick up antigens and migrate into draining lymph nodes to present antigens to naïve T cells for the induction of effector cells. In the elicitation phase, the next exposure of antigen drives antigen presentation cells to initiate effector cell activation and cause inflamed tissue reaction.

The pathogenesis of drug eruption is classified into allergic and non-allergic reactions [13]. In an allergic reaction, the majority of drug eruption is mediated by type IV delayed hypersensitivity reaction, and the importance of this mechanism is proven by commonly used patch testing and lymphocyte stimulation test for the diagnosis [14,15,16,17]. On the basis of the pathogenesis, type IV delayed hypersensitivity reaction consists of two phases, namely, sensitization and elicitation (Figure 1) [9,18]. In the sensitization phase, the exposure to external antigens promotes cutaneous dendritic cell maturation and migration to draining lymph nodes for the induction of antigen-specific memory T cells from naïve T cells. After the establishment of sensitization against the causative drug, the next administration of the drug initiates the elicitation of the immune response and causes cytotoxic cell-mediated immune responses in peripheral lymphoid organs.

In the severe types of drug eruption, cytotoxic immune cells, such as CD8^+^ T cells, NK cells, and NKT cells identify the specific epitopes HLA-drug interaction and produce cytotoxic proteins such as Fas ligand, perforin, granzyme B, and granulysin and provoke epidermal keratinocyte cell death, which is associated with the severe clinical manifestation of SJS/TEN.

## 3. Clinical Sign and Biomarker for Drug Eruption

### 3.1. Intertriginous Eruption as a Clinical Sign for Acute Generalized Exanthematous Pustulosis (AGEP)

Intertriginous drug eruption is an exanthem characterized by the rapid onset after the causative drug administration and occurs in the predilection sites including inguinal folds and axillae [19,20], and is recognized in some cases, but not all, as the mild form of AGEP. As the mechanism of intertriginous drug eruption, type IV delayed hypersensitivity reaction has been postulated by in vitro lymphocyte stimulation test with the causative drugs by using patients peripheral blood mononuclear cells (PBMCs) [19]. The strength of this symptom is easy to identify as the clinical symptoms to predict the development of AGEP. On the contrary, the weakness has been found by a single case report. Therefore, the accumulation of multiple observational studies will be desired to determine how helpful this finding is for the detection of the early stage of AGEP.

### 3.2. Peripheral Blood Eosinophil Count

A previous study showed increased infiltration of eosinophils in the skin and the high frequency of eosinophils in peripheral blood in patients with drug eruption. The presence of eosinophils in peripheral blood differs in each type of drug eruption and the number of eosinophils in patients with drug-induced erythema multiforme is increased, compared with other types of drug eruption [21]. A total of 113 patients were enrolled in this study and an increased eosinophil number reflects an unfavorable clinical behavior, such as liver dysfunction, elongation of a duration of corticosteroid administration and hospitalization, suggesting that eosinophil is closely associated with the severity of drug eruption. The strongness of the peripheral blood eosinophil count is commonly examined in clinics and is easy to examine. On the contrary, the weakness is that this finding is not specific for drug eruption. Peripheral blood eosinophil also increases in atopic dermatitis with a positive correlation with the severity [22,23]. Peripheral blood eosinophil increase in patients with psoriasis and urticaria [24]. From these findings, peripheral blood eosinophil is not specific in drug eruption; however, it might be easy to evaluate the severity of drug eruption.

### 3.3. Serum Eosinophilic Cationic Protein

Eosinophilic cationic protein is a cationic protein derived from eosinophil granulocyte, which is a marker for drug eruption [24]. Serum eosinophilic cationic protein levels were significantly increased in patients with drug eruption compared to healthy subjects. In a mouse experiment, eosinophilic cationic protein increases 3 h after allergen exposure [25], suggesting that this might reflect an early time allergen reaction to the causative agents. Eosinophilic cationic protein does not correlate with the peripheral blood eosinophil count, however, serum eosinophilic cationic protein is increased with the positive correlation with the activated eosinophil in the skin [26]. All 4 cases of drug eruption showed increased serum eosinophilic cationic protein, while all 10 healthy subjects showed low levels of serum eosinophilic cationic protein.

### 3.4. The Presence of CD4^+^CD25^+^ Cell in the Epidermis as a Sign of Desensitization in Fixed Drug Eruption

Desensitization is one of the highlighted issues for clinical aspects in patients with drug eruption. However, there is a limited number of findings to estimate the sign of desensitization. Although fixed drug eruption often shows the presence of CD8^+^ cells in the epidermis, CD4^+^CD25^+^ cells are also increased in the epidermis as a sign of desensitization of fixed drug eruption [27]. Because this was investigated in only one subject, further large number studies will be desired to evaluate the actual impact on the desensitization mechanisms. As a weakness, CD25+ cells do not directly mean regulatory T cells and this could not exclude the possibility of reactive T lymphocytes [28].

### 3.5. miR-18a-5p, miR-124, and miR-214

MicroRNA (miRNA) is a small single-stranded and non-coding RNA molecule and is observed in various living things, such as animals and plants. miRNA is functionally silencing RNA and suppresses post transcription of gene regulation [29]. Circulating miRNAs are released into the blood and have the potential as biomarkers in various diseases [30,31,32]. An increased miR-18a-5p in the skin is observed in patients with TEN [33]. To evaluate the actual impact of miR-18a-5p, transfection of miR-18a-5p induces an increased apoptotic cell in keratinocytes and upregulates caspase-9 activity in the skin in patients with TEN. In addition, miR-18a-5p suppresses B-cell lymphoma/leukemia-2-like protein 10 (*BCL2L10*) gene expression, which is an anti-intrinsic apoptotic molecule. *BCL2L10*-silencing keratinocytes provoke both apoptosis and caspase-9 activity. Furthermore, serum miR-18a-5p increases in patients with TEN with a positive correlation with body surface areas of skin eruption. miR-18a-5p functionally promotes toxic reactions in the pathogenesis and is also a biomarker for patients with TEN. Serum samples were obtained from 8 patients with TEN, 10 patients with SJS, 15 patients with EM minor, and 22 healthy subjects in this study.

Both miR-124 and miR-214 expressions are increased in patients with severe drug eruptions and are reported as possible biomarkers of TEN [34]. The serum concentration of miR-124 shows a positive correlation with the severity of drug eruption and the high scoring of SCORTEN. The miR-214 expression is increased in lesional skin in patients with TEN. Serum samples were obtained from 7 TEN patients, 5 SJS patients, 11 EM minor patients, and 21 healthy subjects in this study. As a limitation, this study was a single-institution study and was a relatively small number investigation.

The actual action of these miRNA in the pathogenesis of drug eruption remains unclear. However, the action of miRNA is considered to be: (1) specific changes in the methylation pattern, (2) post-transcriptional modifications of specific histone proteins, (3) differences in chromatic packing, in addition to (4) the role of the Polycomb and Thritorax proteins, which can be used as markers. Epigenetic changes can modulate gene transcription not to alter DNA sequencing information by histone modification and DNA methylation [35]. miRNA also plays a pivotal role in the regulation of epigenetics [36,37]. Therefore, we speculated that these miRNA might also have an epigenetic modification and/or post-transcriptional modification in the drug eruption.

Actually, miR-18a-5p regulates the post-transcription and metastasis of cancer cells [38]. miR-124 gene contributes to a condensed chromatin structure mediated by histone modification [39], and also suppresses gene transcription [40]. Polycomb protein EZH2 is regulated by miR-124 and miR-214, which act as antitumor effects [41,42] and cell differentiation [43]. Although the contribution of epigenetic modification in drug eruption remains unclear, these epigenetic changes might regulate the development of drug eruption.

### 3.6. Perforin and Granzyme B

**Figure 2:**
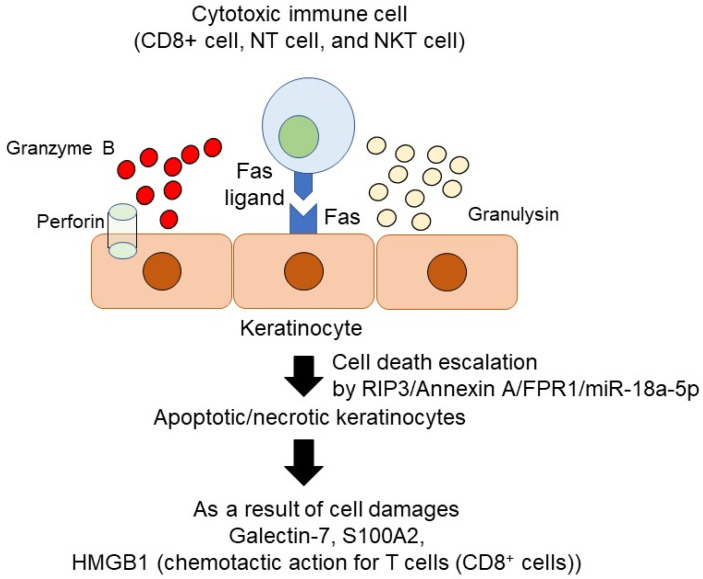
The molecular mechanism of cytotoxic cells mediated keratinocytes damages. There are three key mechanisms, Fas-Fas ligand-mediated cell death, perforin/granzyme B mediated cell death, and granulysin mediated cell death.

For the induction of the cytotoxic activity of immune cells, such as CD8^+^ cells and NK cells, they produce perforin, which is a cytolytic mediator and is released by cytoplasmic granules in these immune cells (Figure 2). Perforin makes the pore in the cell targeted membrane up to 20 nm in diameter and initiates cytotoxic molecules entering into the cytoplasm [44]. Granzyme B is a serine protease that initiates cell apoptosis in a perforin-dependent manner produced by cytotoxic cells [45]. Granzyme B cleaves and activates various caspases, such as caspases 3 and 7, which trigger cell apoptosis [45]. Granzyme B also has many substrates in the nucleus, leading to DNA disruption. In severe cutaneous adverse reactions, serum perforin and granzyme B are increased in patients with SJS/TEN in an early time course [46,47]. Because their importance has been reported in several studies, perforin and granzyme B are reliable markers for SJS/TEN.

### 3.7. Annexin A1 and RIP3

Annexin A1 belongs to the danger-associated molecular patterns (DAMPs) and is released by the cytoplasm of apoptosis cells. Once annexin A1 binds formyl peptide receptor 1 (FPR1) expressed on dendritic cells, this interaction enhances long-term between apoptosis cells and dendritic cells [48]. Annexin A1 is also one of the mediators for keratinocyte death in severe cutaneous adverse drug eruption [49]. The depletion of annexin A1 by antibody treatment impairs cytotoxicity against keratinocytes. Keratinocytes in patients with SJS/TEN highly express FPR1 in the skin. Therefore, annexin A is not only a biomarker and but is also possibly a therapeutic target in SJS/TEN.

In addition, necroptosis is programmed cell death and is involved in various inflammatory diseases [50], and necroptotic cells release damage-associated molecular patterns (DAMPs) and inflammatory cytokines, such as TNF-α. TNF-α stimulates receptor interacting kinase 1 (RIP1) and receptor interacting kinase 3 (RIP3), which are phosphorylated and then form a “necrosome” complex inducing keratinocytes apoptosis [49]. The serum levels of RIP3 and the degree of RIP3 expression in the epidermis are associated with the severity of cutaneous adverse reactions [51]. Fifty-two patients with SJS/TEN, 5 patients with erythema multiforme minor, 19 patients with erythema multiform major, and 53 patients with maculopapular exanthema were enrolled in this study.

### 3.8. Soluble Fas Ligand

Fas ligand is a type-II transmembrane protein belonging to the tumor necrosis factor (TNF) family. After binding the ligand, Fas forms the death-inducing signaling complex (DISC) and is internalized via the cellular endosomal mechanisms and then causes caspase-8 activation into the cytoplasm, eventually leading to DNA and cell membrane damages for the induction of apoptosis. Soluble Fas ligand is detected in the early stages of SJS/TEN [52]. Ordinal drug eruption and healthy subjects showed no increase of soluble Fas ligand in serum. Therefore, soluble Fas ligand is known as a useful biomarker to identify severe cutaneous adverse reactions among patients with drug eruption. The sera of 19 patients with SJS and 16 patients with TEN at 1 or multiple time points were obtained from Japanese multiple hospitals in this study.

### 3.9. Granulysin

Granulysin is a cytolytic and proinflammatory molecule produced by activated cytotoxic T cells and NK cells [53]. Granulysin has cytotoxic activity against tumor cells and other microorganisms by which it influences the negatively charged cell membranes mediated by the positive charge of granulysin (4). Serum granulysin is highlighted as a biomarker in severe cutaneous adverse events. Serum granulysin is significantly increased in SJS/TEN patients, and the immunochromatography examination showed positive results of granulysin in 80% of SJS/TEN patients, with 1 out of 24 patients presenting ordinal drug eruption [54]. Five patients with SJS/TEN, 24 patients with ordinal drug eruption, and 31 healthy subjects were enrolled and collected serum in this study. Another study showed that drug-induced hypersensitivity syndrome/drug reaction with eosinophilia also show high serum levels of granulysin [55]. Twenty-one patients with DRESS and 29 healthy subjects were enrolled in this study.

### 3.10. High Mobility Group Box 1 Protein (HMGB1)

Likewise histone, HMGB1 is the important chromatin protein and interacts with nucleosomes to regulate gene transcription [56]. In addition, HMGB1 has been recognized as a danger signal involved in the pathogenesis of various inflammatory diseases, malignancies, and injury [57,58,59]. On the contrary to these functional roles, HMGB1 is also useful as a biomarker in various diseases [60,61]. HMGB1 is noticed in serum in patients with severe cutaneous adverse reactions [62,63]. Serum HMGB1 levels in patients with SJS/TEN from 7 days before the onset and 21 after the onset of diseases were significantly increased compared with healthy controls and patients with ordinal drug eruption [62]. Twenty-two healthy subjects and 13 patients with SJS/TEN were enrolled in this study and the sera were obtained from multiple institutions. In addition to the high serum level of HMGB1, HMGB1 concentration is also increased in bullous fluids in patients with SJS/TEN [63]. This study was conducted by three independent SJS/TEN patient cohorts and was then brought together for the purpose of this study. Nine patients with SJS/TEN were enrolled in Nevirapine-induced cases study, 73 patients with SJS/TEN were enrolled in a Taiwanese study to evaluate the serum HMGB1 concentration. In addition, 22 patients with SJS/TEN were enrolled in a Spanish study to evaluate the bullous concentration of HMGB1.

### 3.11. Thymus and Activation-Regulated Chemokine (TARC)

TARC is a chemokine belonging to the CC chemokine family, namely CCL17, and is produced by immune cells in allergic diseases [64,65]. TARC binds to CCR4, which is dominant in Th2-dominant immune cells for their chemotaxis [66]. TARC levels in serum in patients with drug eruption can predict systemic inflammation and disease severity [67]. Serum TARC levels positively correlated with the severity of cutaneous inflammation, such as white blood cell count, C-reactive protein, and the severity of the score. Twelve patients with DRESS/DIHS, 18 patients with maculopapular exanthema, and 46 patients with erythema multiforme were enrolled and SJS/TEN patients were eliminated from this study, indicating that TARC might not reflect the actual severity of drug eruption. In addition, TARC is not specific in drug eruption and is also increased in other inflammatory skin diseases, such as atopic dermatitis [65,68] and psoriasis [69].

### 3.12. Th17

IL-17-producing cells are involved in various inflammatory diseases, such as psoriasis [70,71,72,73,74], asthma [75,76,77,78,79], and atopic dermatitis [80,81,82], and contribute to the development of host defense against microorganisms [83,84,85,86]. As the importance of IL-17-producing cells in the human body, IL-17-targeted biologics treatment is proven by the current advancement of this field. Th17 cells are known as the main source of IL-17 in humans, and the percentages of Th17 cells are increased in DIHS (10 days after the onset) and SJS/TEN (2–6 days after the onset) as compared to normal subjects and maculopapular drug eruption (2–6 days after the onset) [87]. Fifteen patients with maculopapular type, 15 patients with erythema multiforme, 1 patient with SJS, 1 patient with TEN, and 4 patients with DIHS were enrolled in this study. During the clinical course of DIHS, the frequencies of Th17 cells are gradually decreased. Peripheral blood Th17 cells migrate into the skin on day 12–21 after the onset. This information suggests to us that Th17 cells could not detect at the very early time point in the clinical time course in patients with severe type drug eruption. In another study, the frequency of Th17 did not differ significantly between SJS/TEN patients and healthy controls, possibly due to the missing time course point [88]. Peripheral blood mononuclear cells were obtained from SJS/TEN patients (acute stage: *n* = 3, resolution stage: *n* = 7) and 24 healthy subjects. Therefore, Th17 cells might not be useful for the biomarker in severe type drug eruption in an early time point, however, this might be a compliment for the lack of information of other important biomarkers for cutaneous adverse events such as SJS/TEN. As the importance in the pathogenesis, Th17 might also promote an additional cutaneous inflammatory response in severe type drug eruption, because Th17 enhances subsequent inflammatory immune responses. Consistently, an increased frequency of Th17 cells has been reported in AGEP [89]. Peripheral blood mononuclear cells were obtained from 3 patients with AGEP in this study. Therefore, it is speculated that IL-17 and IL-22 cooperatively stimulate keratinocytes to enhance IL-8 production, leading to the contribution of neutrophil infiltration in the lesional epidermis of AGEP. In addition, psoriasiform drug eruption mimicking as psoriasis vulgaris is sometimes observed in the treatment of some biologic agents, such as TNF or IL-17 inhibitors, which are also involved in Th17 in the skin [90,91], suggesting that Th17 cells play some role in the pathogenesis of drug eruption not in the main but in a part of the stream. As for the weakness, Th17 is observed in various other inflammatory skin diseases, such as psoriasis and atopic dermatitis; therefore, Th17 is not a specific biomarker of drug eruption.

### 3.13. Serum Galectin-7

Galectin-7 is a β-galactoside-binding protein family and is expressed in epithelial cells [92]. Keratinocytes are one of the major cells to express galectin-7, which is closely associated with keratinocyte survival and growth [92]. Galectin-7 has been investigated and used to discover biomarkers in various diseases [93,94,95,96,97,98]. Galectin-7 has examined the possible role of the biomarker in severe cutaneous adverse events [99]. The causative drug was determined from lymphocyte stimulation test in SJS and TEN. The supernatant of lymphocyte stimulation test using peripheral blood mononuclear cells taken from these patients after 1 day was collected and subjected to the mass spectrometry analysis. Galectin-7 was identified as the possible proteins as a biomarker for SJS/TEN and the serum concentration of galectin-7 was increased in the 24 patients with SJS/TEN compared with that in 8 healthy subjects. The weakness of the galectin-7 is not a specific marker of drug eruption, and is also seen in other inflammatory skin diseases, such as atopic dermatitis [100] and UV radiation [101], while it is decreased in psoriasis [102].

### 3.14. S100A2

S100 calcium-binding protein A2 (S100A2) is a member of the EF-hand motif family S100 [103] and is recognized as a tumor-suppressive role [104]. S100A2 is mainly expressed in epithelial cells and contributes to various cell functions [105], and is recognized as a biomarker in oncology fields [106,107,108,109,110]. However, a recent study identified that S100A2 is a possible biomarker in severe cutaneous adverse events [111]. Forty-one cases of macular type, 14 cases of maculopapular type, and 9 cases of severe types of drug eruption (7 cases of Stevens–Johnson syndrome and 2 cases of toxic epidermal necrolysis) were enrolled in this study. This study was conducted by using telaprevir or trichloroethylene, which causes high frequently cutaneous adverse events. Telaprevir or trichloroethylene exposed keratinocytes highly enhance S100A2 proteins by microarray analysis and S100A2 is increased in the skin in patients with severe type drug eruption. However, S100A2 is not a specific biomarker because S100A2 is also upregulated in atopic dermatitis and psoriasis patients.

### 3.15. The Difference of Generalized Bullous Fixed Drug Eruption Compared with SJS and TEN

Generalized bullous fixed drug eruption is a kind of particular clinical manifestation of fixed drug eruption and is characterized by wide-spreading blisters and erosions, which mimic the clinical characteristics of SJS/TEN. Patients with generalized bullous fixed drug eruption exhibit shorter latent periods and less mucosal involvement, however, more eosinophil infiltration and dermal melanophages. Twenty-three cases of GBFDE were enrolled in this study [112], and increased infiltration of CD4^+^Foxp3^+^ regulatory T cells in the dermis and reduced intraepidermal granulysin^+^ cell infiltration were observed in generalized bullous fixed drug eruption. In addition, the serum granulysin in generalized bullous fixed drug eruption was significantly lower than that in SJS/TEN. Therefore, it might be helpful to gain a clue of the diagnosis of generalized bullous fixed drug eruption by evaluating the serum granulysin in addition to these histological analyses to distinguish between generalized bullous fixed drug eruption and SJS/TEN.

### 3.16. T Cell Receptor-Cβ1 (TCR-Cβ1) Gene Rearrangement for Pseudolymphoma CD30^+^ Large Cell Transformation

Lymphocytes acquire the diversity of antigen recognition through T-cell receptor gene rearrangement during cell differentiation. On the contrary, malignant lymphoma tumor cells exhibit a monoclonal change of tumor growth and T-cell receptor rearrangement [113,114,115]. Therefore, a monoclonal T-cell receptor gene band is observed in lymphoma cells. This method is commonly used in the clinical aspect to distinguish between benign and malignant lymphoid tumors. Pseudolymphoma is one of the lymphoid cell proliferation diseases and is characterized by solid papules and nodules [116,117], which mimic lymphoma and lymphomatoid papulosis [118,119]. The clinical manifestation is the solid popular with the presence of CD30^+^ large transformed atypical cells in the skin. Although the pathogenesis of CD30^+^ pseudolymphoma remains unclear, the drug administration is one of the triggers for this cutaneous reaction [120,121,122] and the reactive T cells are overstimulated during the causative agent’s treatment and as a result, the pseudolymphoma might cause after a long duration of the incubation period [120]. As a limitation, these are case studies. For clinicians, lymphomatoid papulosis is a differential diagnosis that is clearly distinguished by the absence of monoclonal T-cell receptor-Cβ1 gene rearrangement in the lesional skin [121].

### 3.17. Metalloproteinase

A metalloproteinase is any protease enzyme mediated by a catalytic mechanism involved in metals. Metalloproteinases play a crucial role in various cell functions, such as cell migration [123]. Previous studies have identified the relationship between drug eruption and metalloproteinases. A high amount of MMP2 was observed in TEN blister fluid [124]. Blister fluid from 6 patients with TEN patients was compared with 3 other blistering conditions. Six patients with bullous pemphigoid, 13 patients with second-degree burn, and 3 patients with suction blister were examined in this study. In addition, the presence of MMP2, MMP9, and MMP11 was observed in the skin in patients with SJS/TEN [125,126]. One study was performed, where skin biopsies were taken from the edge of the blisters of 2 patients with TEN, 3 patients with SJS, and 2 healthy subjects [125], and another study conducted skin biopsied taken from 8 patients with erythema multiforme and 6 patients with SJS/TEN [126], respectively. MMP2 and MMP9 are not specific in drug eruption. MMP2 is increased in psoriasis [127] and MMP9 is increased in atopic dermatitis [128], psoriasis [129], and urticaria [130]. On the contrary, MMP11 has currently not been reported in inflammatory skin diseases, such as psoriasis and atopic dermatitis. Although further investigation is necessary, MMP11 might be a specific biomarker in durg eruption.

### 3.18. Prognostic Biomarkers for SJS and TEN

Since fatal cases and long-term complications are observed following cutaneous adverse reactions, the predictive tools for these cases are helpful for clinicians. Severity-of-illness score for toxic epidermal necrolysis (SCORTEN) is a representative severity scoring for SJS/TEN to estimate their prognosis, based on seven independent clinical parameters; age > 40 years, the presence of malignancy, tachycardia > 120/min, body surface area affected > 10%, serum urea > 28 mg/dL, serum glucose > 250 mg/dL, serum bicarbonate < 20 mEq/L [131]. SCORTEN represents a specific severity-of-illness score for TEN, which was highly accurate in predicting mortality (19.6% predicted, vs. actual 20% mortality).

There are several reported biomarkers to predict their prognosis or complication following cutaneous adverse reactions. An allopurinol-related severe cutaneous adverse reaction investigative study showed that poor renal function is significantly associated with the delayed clearance of plasma oxypurinol, in addition to the high risk of severe cutaneous adverse reaction. High plasma levels of oxypurinol and granulysin are also related to the high mortality of allopurinol-SJS/TEN [132].

Low serum bicarbonate less than 20 mmol/L has a significant association with mortality, the odds ratio in the patients with TEN having low serum bicarbonate being 40 times higher than those without patients [133]. Sixteen patients with TEN were enrolled in this study. Serum bicarbonate is an easy examination from peripheral blood; however, this simply reflects the acidosis in the body, suggesting that low serum bicarbonate is expected to be seen in other skin diseases to cause metabolic acidosis, such as burn [134].

Endocan is a potential immunoinflammatory marker, and high endocan levels in serum of endocan were observed [135]. Seven patients with SJS/TEN were enrolled in this study. SJS/TEN patients show higher endocan levels compared with normal subjects in addition to a positive correlation with the severity of SCORTEN. Endocan is evaluated by peripheral blood serum level, and it is easy to obtain the sample. However, endocan is also not a specific marker and is also increased in atopic dermatitis in serum and lesional skin [136], psoriasis [137], and angioedema [138].

IL-13/IL-15 is known as a biomarker in SJS/TEN patients. IL-13 positive cells in the lesional SJS/TEN skin specimens showed significantly higher levels compared with that in EM specimens [139]. Eight patients with EM, 6 with SJS/TEN, and 3 healthy subjects were enrolled in this study. In addition, increased expression of IL-13 and IL-15 in the plasma samples was observed in SJS/TEN patients [140]. A total of 12 patients with SJS/TEN were analyzed in this study. Furthermore, IL-15 was associated with mortality in SJS/TEN patients [141]. A total of 155 patients with Stevens-Johnson syndrome/TEN were enrolled in this study. However, these cytokines are not specific in drug eruption. IL-13 is increased in atopic dermatitis [142]. In addition, IL-15 is also increased in bullous diseases [143], T cell lymphoma [144], and psoriasis [145].

## 4. The Summary of Biomarkers Depending on the Time Course of SJS and TEN

Table 1 summarises each biomarker in drug eruption. Biomarkers in serum well reflect the systemic condition and are samples that are easy to collect, subjected to the development of further examination. However, clinicians should keep in mind that there are better time course points to evaluate these biomarkers. Soluble Fas ligand, granulysin, granzyme B, and perforin are increased in the early time point during the onset of SJS/TEN, and they are rapidly decreased after several days of disease onset. Soluble Fas ligands increased 2–6 days before the onset of SJS/TEN and are not detected the day before the onset of diseases [52]. Granulysin also showed a similar time course and increased 4 days before and 2 days after the onset, and is not detected three days after the onset [146]. Perforin and granulysin are increased within 1 day after the onset [47], however, granzyme B is only detected around 8 days after the onset [55].

Eosinophils in peripheral blood and tissues and eosinophil cationic protein are also increased in patients with severity, however, they are also observed in psoriasis and acute urticaria [21,24]. A high number of eosinophils in peripheral blood is helpful for the diagnosis of drug eruption however, eosinophilia is also observed in bullous pemphigoid, which becomes a differential diagnosis of SJS/TEN. In addition, there is no additional study to determine the importance of eosinophil cationic protein as a biomarker of cutaneous adverse reactions. Serum miR-18a-5p and miR-124 are increased in patients with severe cutaneous adverse events drug eruption, however, the timing of blood sample collection and specificity are not defined [33,34], and it remains unclear whether these are derived from tissue damage reaction or the possible pathogenesis. Galectin-7 concentration in serum increases in SJS/TEN patients before treatment, however, this is not a specific finding in drug eruption and is also upregulated in other inflammatory diseases as a result of tissue damage [100]. HMGB1 is constitutively increased in SJS/TEN and this significantly increases compared with other types of drug eruption and healthy subjects [62,63], which means an increase in the value of HMGB1 does not necessarily reflect the clinical time course point, however, HMGB1 is not a specific marker for drug eruptions [147,148].

The bullous fluid is one of the samples to identify the local inflammatory reaction to accumulate the inflammatory response in the site. Indeed, the bullous fluid contains above early time course biomarkers, such as soluble Fas ligands [63] and HMGB1 [63], however, they only reflect local skin site inflammation, depending on the sampling technique and the degree of the local site inflammation.

Histological examination is a basic standard method and might reflect the time course. However, there has a limitation to conduct objective evaluation, such as the degree of positive cells. Traditionally, the presence of dyskeratotic cells in the epidermis is helpful as a clue for the diagnosis of severe type drug eruption. In addition, histological analysis of S100A2 and HMGB1 reflects the severity of drug eruption [62,111], however, they could not reflect the specificity of drug eruption, because they are increased in other inflammatory skin diseases, such as atopic dermatitis and psoriasis. In addition, some candidate biomarkers show an unspecific increase in the serum following the specific causal drugs not related to SJS and TEN. For instance, antiretroviral therapy itself significantly increases the serum level of HMGB1 following the treatment [149]. On the contrary, some specific disease conditions also affect the degree of these biomarkers. An increased serum HMGB1 level is observed in febrile seizures [150] and end stage renal disease [151]. S100A2 also reflects the severity of atopic dermatitis, as a result of keratinocyte damages. miR-214 is increased in severity type drug eruption, however, the specificity is not clarified [34]. Although RIP3 is driven under a more specific condition, this could not also exclude other inflammatory disease conditions, such as psoriasis [50].

As a limitation of biomarkers regarding the correlation between skin tissue reaction and serum/plasma and blister fluid levels of the biomarkers, skin tissue reaction does not always reflect the serum levels of biomarkers. Therefore, systematic analysis is used to clarify the interaction between local skin tissue reaction and systemic reaction by peripheral blood and serum.

## 5. Conclusions

This review summarized the clinical signs, diagnostic tools, and biomarkers for drug eruption, in addition to a recent update of this field research. The presence of biomarkers differs in each time course of severe type drug eruption, and we should keep in mind that the disease onset date-based examination of biomarkers is helpful for the diagnosis of SJS/TEN with the combination of histological and serum examination. There are several useful markers for SJS/TEN diagnosis, however, specificity is also the next problem for the diagnosis of SJS and TEN. In the current options, soluble Fas ligands and granulysin are some of the most reliable tools for the diagnosis of SJS/TEN. However, if we face the clinical cases with a suspected diagnosis of SJS/TEN and there is a limitation to evaluate soluble Fas ligands and granulysin, especially due to the delayed sampling, other biomarkers might be useful for the detection of SJS/TEN to predict the future clinical course in these patients of drug eruption. As the prognostic estimation tool for SJS/TEN patients, SCORTEN is a better tool in addition to other biomarkers, such as serum bicarbonate, endocan, and IL-15. These combination examinations will be helpful to determine the therapeutic approach for drug eruption.

## Figures and Tables

**Table 1:** The summary of biomarkers and relationship with the pathogenesis.

Biomarker	Type of Drug Eruption	Mechanism	Direct or Indirect Action	Detection
Eosinophil count	General drug eruption	Immune activation	Indirect action	Peripheral blood
Eosinophilic cationic protein	General drug eruption	Unknown	Indirect action	Serum
CD4^+^CD25^+^ cell	A sign of desensitization of fixed drug eruption	Immune activation	Direct action	Skin
miR-18a-5p	TEN	Immune activation	Direct action	Skin
miR-124	TEN	Unknown	Unknown	Serum
miR-214	TEN	Unknown	Unknown	Skin
Perforin	SJS/TEN	Immune activation	Direct action	Serum
Granzyme B	SJS/TEN	Immune activation	Direct action	Serum
Fas ligand	SJS/TEN	Immune activation	Direct action	Serum, bullous fluid
Granulysin	SJS/TEN	Immune activation	Direct action	Serum
HMGB1	SJS/TEN	Tissue damage	Indirect action	Serum, bullous fluid, skin
TARC	General drug eruption	Immune activation	Indirect action	Serum
Th17	DIHS, AGEP, SJS/TEN	Immune activation	Indirect action	Peripheral blood
Galectin 7	SJS/TEN	Tissue damage	Indirect action	Serum
S100A2	SJS/TEN	Tissue damage	Indirect action	Skin
Annexin A/FPR1	SJS/TEN	Immune activation	Direct action	Skin
RIP3	SJS/TEN	Immune activation	Direct action	Serum, skin
IL-13/IL-15	SJS/TEN	Immune activation	Direct	Serum, skin
MMP2, MMP9, MMP11	SJS/TEN	Immune activation	Direct action	Skin
